# Supplementary material for: Advances in deep reinforcement learning enable better predictions of human behavior in time-continuous tasks
Source: PLoS One. 2025 Dec 4;20(12):e0338034. doi: 10.1371/journal.pone.0338034 (PMC12677501; doi:10.1371/journal.pone.0338034)

S1 Fig. Visualization of human time series and generated features. Visualization of the original and preprocessed time series of a randomly selected subject for the actions 'fire' and 'right', as well as the original and preprocessed time series of features generated by Ape-X representing the neurons corresponding to the actions 'fire' and 'right' during the first session of Space Invaders.

(A) Time series of features (Q-values) generated by Ape-X.

(B) Preprocessed time series of features generated by Ape-X, smoothed with a Gaussian kernel with FWHM = 0.79 seconds.

(C) Binary time series of the subjects' actions.

(D) Preprocessed time series of the subjects' actions, smoothed with a Gaussian kernel with FWHM = 0.79 seconds.

(E) Time series of human motor responses predicted by the GLM with features generated by Ape-X.

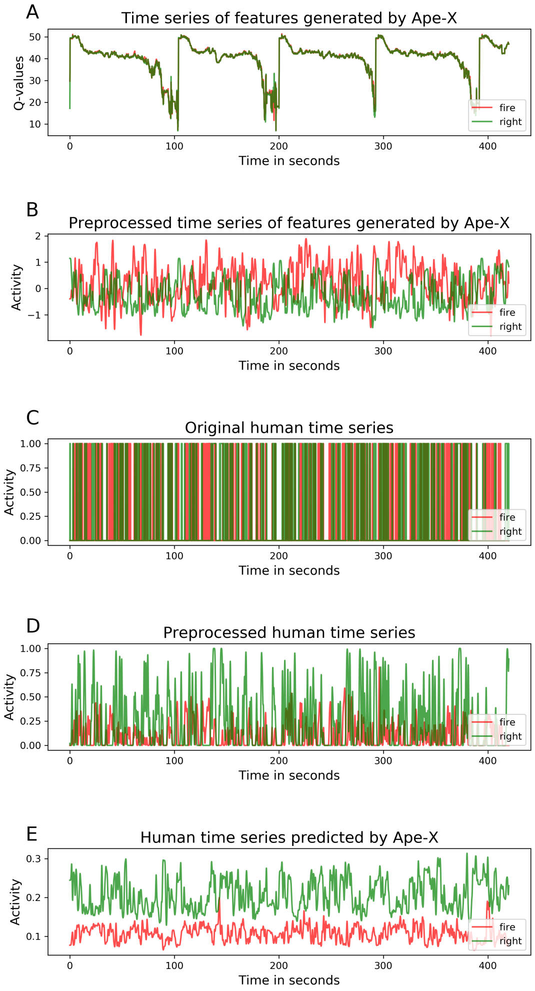

Supplement: S1 Fig — (PDF) [file pone.0338034.s001.pdf]
