# Supplementary material for: Advances in deep reinforcement learning enable better predictions of human behavior in time-continuous tasks
Source: PLoS One. 2025 Dec 4;20(12):e0338034. doi: 10.1371/journal.pone.0338034 (PMC12677501; doi:10.1371/journal.pone.0338034)
Supplement: S6 Text — (PDF) [file pone.0338034.s007.pdf]

S6 Text. Incorporating human reaction times into prediction. The response time of humans varies across individuals and is influenced by numerous factors, but averages around 250 ms for visual stimuli [1]. This corresponds to 11 frames at the temporal resolution of our experiment, which is 45 Hz, whereas a DQN typically has response times of only 1-4 frames. Therefore, we investigated a post-adjustment of the time series of humans and DQNs by shifting the human time series by 7 frames. However, this did not lead to a significant improvement in predictive power, contrary to expectations.

Prediction accuracy, measured as the Pearson correlation coefficient, with subsequent consideration of human response times. We shifted the human time series by 7 frames to align with the time series generated by the DQNs. The time series were smoothed using a Gaussian kernel with FWHM= 0.79 **seconds.**

	**Breakout**	**Space Invaders**	**Enduro**
Baseline DQN	0.16	0.20	0.31
Ape-X	0.25	0.18	0.37
SEED	0.32	0.28	0.48

References

[1] Jain A, Bansal R, Kumar A, Singh K. A comparative study of visual and auditory reaction times on the basis of gender and physical activity levels of medical first year students. International Journal of Applied and Basic Medical Research. 2015;5:124–127. doi:10.4103/2229-516X.157168. PMID:26097821.
